# Mechanophore‐Based Multichannel Colorimetric Force Sensors: Ultrasound‐Driven Stress and Strain Quantification

**DOI:** 10.1002/advs.77607

**Published:** 2026-09-08

**Authors:** Seungo Baek, Jeong Hoon Rhee, Skylar K. Osler, Minsoo P. Kim, Shyuan Cheng, Leonardo P. Chamorro, Hyunhyub Ko, Maxwell J. Robb, Gun Kim

**Affiliations:** ^1^ School of Civil and Environmental Engineering Nanyang Technological University Singapore Singapore; ^2^ Department of Structural Engineering Research Korea Institute of Civil Engineering and Building Technology Gyeonggi‐do Republic of Korea; ^3^ Division of Chemistry and Chemical Engineering California Institute of Technology Pasadena California USA; ^4^ Department of Chemical Engineering Sunchon National University Suncheon Republic of Korea; ^5^ Department of Mechanical Engineering University of South Carolina Columbia South Carolina USA; ^6^ Department of Mechanical Science and Engineering University of Illinois at Urbana‐Champaign Urbana Illinois USA; ^7^ School of Energy and Chemical Engineering Ulsan National Institute of Science and Technology (UNIST) Ulsan Republic of Korea

**Keywords:** focused ultrasound, force visualization, mechanochromism, mechanophores, stress‐strain quantification, structural sensor

## Abstract

Mechanophores with mechanochromic properties have attracted significant attention as force‐responsive materials for structural sensing. However, their applications remain mainly limited to qualitative force visualization, as direct and quantitative extraction of mechanical parameters such as stress and strain from mechanochromic responses has not yet been established in bulk polymer systems. Here, we present a mechanophore‐based polymer sensing platform capable of selectively quantifying localized stress or strain through multichannel mechanochromic responses. Two mechanophores with distinct activation thresholds were crosslinked within a single polydimethylsiloxane (PDMS) matrix, enabling differentiated force‐dependent color changes and threshold‐resolved mechanochromic responses under spatiotemporally resolved focused ultrasound (FUS) stimulation. RGB‐based image analysis combined with deep‐learning‐assisted segmentation enabled quantitative stress estimation, while embedded tracer microbeads and particle tracking velocimetry (PTV) enabled strain quantification within the activated regions. This integrated framework establishes a quantitative mechanochromic sensing strategy capable of extracting both stress and strain from optical signals in a spatially resolved and reversible manner, demonstrating the potential of mechanophore‐based polymers as quantitative, electronics‐free sensing platforms for next‐generation structural health monitoring applications.

## 1 Introduction

Identifying stress and strain is essential for understanding material performance and maintaining structural safety across a wide range of applications, from civil infrastructure to biomedical devices [1, 2, 3, 4, 5, 6, 7]. These mechanical parameters govern how structural components respond to external stimuli, yet precise in‐situ analysis remains challenging or not yet practically feasible [8, 9, 10, 11]. Conventional techniques provide global force measurements but do not provide sufficient resolution needed to capture localized responses, particularly near internal defects, or interfaces where force gradients are most significant. To tackle this challenge, researchers have been exploring the potential of mechanophores as self‐reporting sensors for visualizing force in polymeric materials [12, 13, 14, 15, 16, 17, 18, 19]. These molecules can be seamlessly embedded into diverse polymeric systems, where they trigger a variety of chemical reactions, including directly visible mechanochromic transformations [20, 21, 22, 23, 24, 25, 26, 27]. Among existing mechanophores, naphthopyrans (NPs) are notable for their ring‐opening reactions upon external stimuli, producing intensely colored merocyanine dyes and have been widely developed due to their excellent mechanochromic properties [28, 29, 30, 31, 32, 33, 34, 35, 36]. Moreover, NPs can be structurally tuned to exhibit different activation thresholds, offering the possibility of multichannel colorimetric responses within a single material system when subjected to varying mechanical inputs [28, 37]. This tunable mechanochromic behavior makes NPs particularly attractive for stress and strain sensing, as it enables spatially distributed, visually interpretable mechanical readouts within structurally complex materials. However, establishing differentiated and quantitative mechanochemical responses within a single bulk solid polymer remains a longstanding challenge in polymer mechanochemistry [29, 38].

To transition mechanophores from visualization tools to practical stress and strain sensing components, a quantitative and reproducible correlation between their mechanochromic responses and stress or strain must be established. Such a correlation would enable real‐time mapping of force distribution within materials, facilitating applications in structural diagnostics and health monitoring. Previous studies have attempted to address this by applying global mechanical forces, such as uniaxial tension or compression, to mechanophore‐embedded polymer systems [33, 39, 40, 41, 42, 43, 44, 45, 46]. However, these approaches are fundamentally limited by the difficulty of localized force control, which hinders a clear correlation between applied force and resulting stress or strain [47, 48]. In addition, they do not readily allow differentiated activation of multiple mechanophores within a single solid matrix and fail to enable quantitative stress/strain estimation. As a result, they fall short of providing the reliability and calibration required for practical sensor applications. Without spatial control or localized force delivery, it becomes difficult to interpret color changes in terms of reliable mechanical quantities. To leverage the full potential of mechanophores as reliable sensing components, it is essential to investigate their mechanochromic behavior under localized, well‐defined loading conditions. Importantly, even under such conditions, existing studies have primarily interpreted mechanochromic responses qualitatively, without establishing a direct framework for extracting quantitative mechanical parameters such as stress and strain.

Recently, researchers have begun to explore focused ultrasound (FUS) as a non‐contact, noninvasive tool for applying localized mechanical force to mechanophore‐embedded materials [49, 50, 51]. Unlike conventional global loading approaches (e.g., MTS [52, 53]), FUS offers spatiotemporal precision [54], enabling activation of mechanochromic responses only within defined regions (i.e., the focal spot). These studies provide proof‐of‐concept evidence that localized forces can elicit visible color changes, showing the feasibility of spatially resolved mechanochromic activation under localized loading conditions. Despite these advances, a critical gap still remains: the lack of a quantitative framework to correlate localized color intensity with mechanical parameters. This limitation hinders the development of mechanophore‐based systems as practical sensors. As interest grows in advanced diagnostics across areas such as structural health monitoring [8], and biomedical engineering [55, 56], the need for accurate, localized, and noninvasive mechanical sensing continues to increase. However, existing approaches have yet to deliver calibrated platforms capable of extracting mechanical values from colorimetric signals under localized loading conditions [49, 57, 58]. Addressing this challenge is critical to realize the full potential of mechanophores–not only as visualization tools, but as quantitative, real‐time sensors for complex structural components.

**FIGURE 1:**
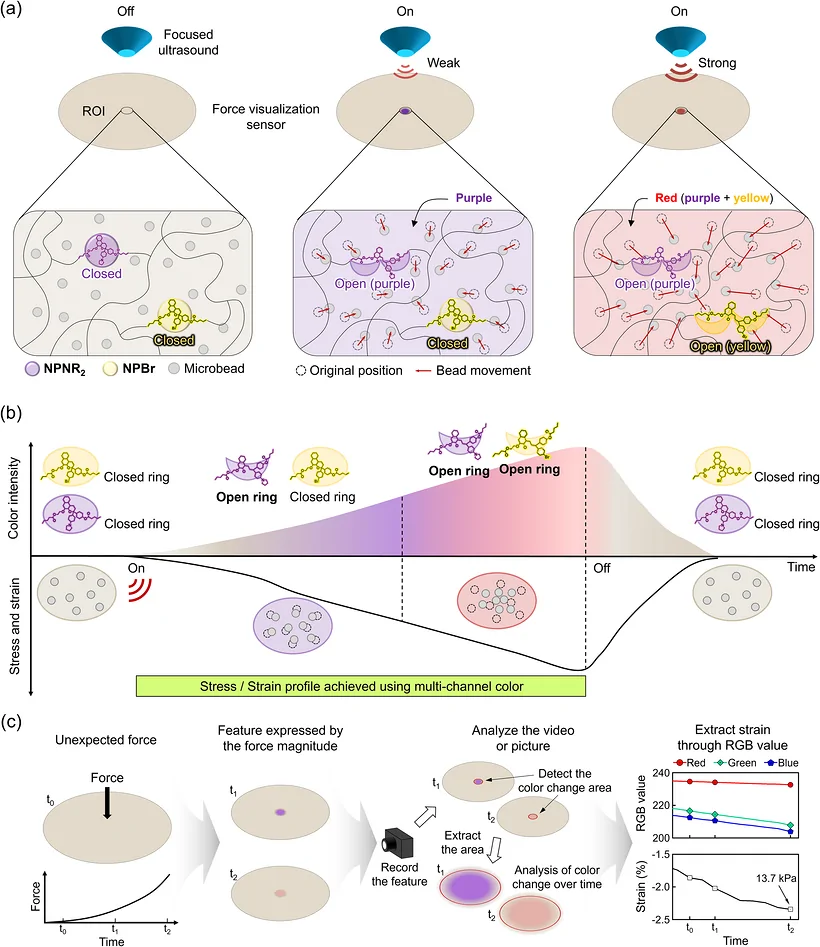
Schematic of the multichannel mechanophore‐based polymer sensor for stress/strain visualization. (a) Spatiotemporally controlled mechanical force is applied via FUS to a PDMS matrix embedded with two mechanochromic mechanophores – **NPNR_2_** (purple) and **NPBr** (yellow)–along with silver‐coated microbeads. Depending on the magnitude of the applied acoustic radiation force, the mechanophores activate sequentially, producing distinct color changes. (b) Schematic illustration of force‐dependent, time‐evolving mechanophore activation during FUS exposure. As the radiation force increases gradually over time, **NPNR_2_** (purple) is first activated above a lower threshold (I_SPTA_ > 867 W cm^−^^2^), followed by **NPBr** (yellow) activation at a higher force level (I_SPTA_ > 1171 W cm^−^^2^). Each mechanophore undergoes a ring‐opening reaction at its characteristic threshold, resulting in a sequential color shift from purple to red (purple + yellow). Upon cessation of force, all mechanophores return to their closed forms, confirming the reversible behavior. (c) RGB profiles over time are analyzed to quantify stress and strain values, establishing direct correlations between color intensity and mechanical deformation.

In this study, we introduce a multichannel mechanophore‐based polymer system capable of quantitatively visualizing stress and strain under spatiotemporally applied force via FUS (Figure 1a). Two NP mechanophores, **NPNR_2_** and **NPBr**, were synthesized to exhibit distinct activation thresholds coupled to unique colorimetric outputs (purple and yellow, respectively) (Figure 1b). These mechanophores were embedded into a polydimethylsiloxane (PDMS) matrix, either individually or in combination, to enable tunable multicolor responses as a function of applied force magnitude. Furthermore, this system enables precise identification and differentiation of the activation thresholds of each mechanophore within a single crosslinked polymer matrix, which is essential for enabling multichannel quantitative sensing. Localized, spatiotemporally controlled force was applied only to targeted sample regions using a custom‐built FUS setup. Under this localized loading condition, the system enables differentiated dual‐threshold activation within a single crosslinked PDMS matrix. A deep‐learning model based on Mask R‐CNN [59] was used to detect and quantify mechanochromic regions from optical recordings, enabling estimation of stress via color intensity and affected area. Simultaneously, microbeads embedded within the PDMS were tracked using PTV [60] to generate strain profiles under FUS activation (Figure 1c). This integrated platform enables direct and quantitative extraction of stress and strain from multichannel mechanochromic responses in a spatially resolved manner. Collectively, these results establish mechanophore‐embedded polymer as quantitative sensing systems rather than qualitative visualization tools, providing a foundation for practical applications in structural health monitoring.

## 2 Results and Discussion

### 2.1 Design of Mechanophore‐Embedded Polymer Systems for Multichannel Force Visualization

**FIGURE 2:**
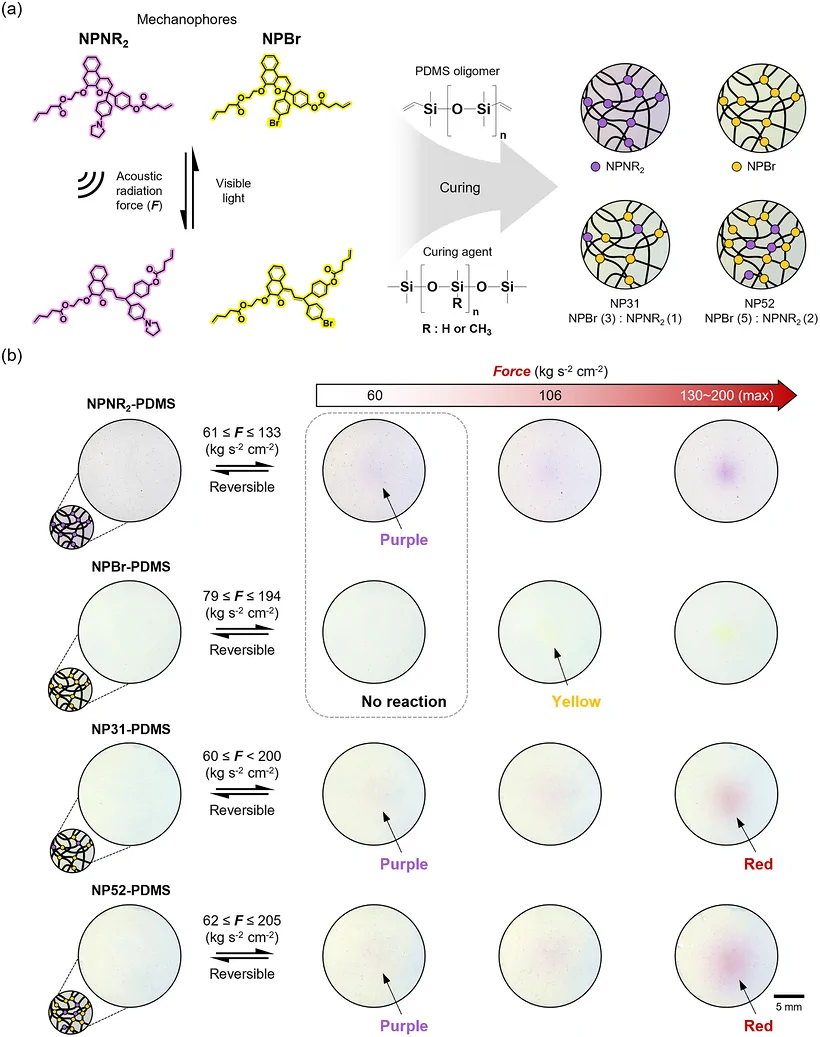
Mechanophore‐integrated PDMS networks and force‐responsive multicolor activation. (a) Schematic of the synthesis strategy for PDMS networks incorporating either single (**NPNR_2_**, **NPBr**), or dual (**NP31**, **NP52**) mechanophore systems. The molar ratio of **NPNR_2_** (purple) to **NPBr** (yellow) is varied to tune force‐dependent colorimetric responses. (b) Examples of digital images showing color changes in mechanophore‐PDMS samples under FUS activation. At a relatively low radiation force (∼60 kg s^−2^ cm^−2^), a distinct purple coloration appeared only in the **NPNR_2_** sample, while **NPBr** remained colorless, confirming the lower activation threshold of **NPNR_2_**. As the force increased (∼61 < *F* < 133 kg s^−2^ cm^−2^), **NPNR_2_** exhibited a reversible purple color, whereas **NPBr** responded only at higher forces (∼79 < *F* < 194 kg s^−2^ cm^−2^), generating a yellow hue. In dual‐mechanophore composites (**NP31** and **NP52**), a force‐dependent transition from purple to red was observed due to sequential activation and spectral overlap of both mechanophores. Compared to **NP31** (∼60 < *F* < 200 kg s^−2^ cm^−2^), **NP52** (∼62 < *F* < 205 kg s^−2^ cm^−2^) exhibited a deeper red hue owing to its higher **NPNR_2_** content (∼14.3% more than **NP31**), enabling a more pronounced multichannel color response. Within the selected force ranges, all color changes were reversible upon force removal.

To achieve multichannel mechanochromic sensing, we designed a series of PDMS samples incorporating mechanophores with different activation thresholds (Figure 2a). Specifically, we synthesized two NP derivatives **NPBr** and **NPNR_2_** (Figure S1). Upon mechanical activation, **NPBr** yields a yellow merocyanine dye, while **NPNR_2_** produces a purple merocyanine [33], enabling visually distinguishable color changes corresponding to different levels of force. These differences are consistent with predictions from density functional calculations using the constrained geometries simulate external force (CoGEF) technique (Figure S1b). The mechanochromic response originates from a force‐induced, reversible 6π‐electrocyclic ring‐opening reaction of the naphthopyran units [28, 37]. Upon mechanical loading, the mechanophores undergo a chemical reaction, converting a closed‐ring to an open‐ring structure (Figures 1b and S1a). This transition significantly extends the *π*‐conjugation system across the molecule, shifting the absorption spectrum into the visible range and producing a distinct color change (Figure 2b). For example, at a relatively low radiation force (∼60 kg s^−2^ cm^−2^), a noticeable purple coloration was observed in the **NPNR_2_** sample, whereas no visible color appeared in **NPBr**, confirming a relatively lower activation threshold for **NPNR_2_**. The absence of yellow confirms that the degree of conjugation directly correlates with the observed optical properties. Upon release of mechanical force, the molecules revert to their thermodynamically stable closed‐ring form, restoring the original conjugation length and color. This reversible modulation of π‐conjugation enables dynamic and repeatable color changes in response to force. Each mechanophore was covalently embedded into a PDMS matrix via crosslinking to form elastomeric disks (5 cm in diameter and approximately 0.5 cm in thickness). In addition to single‐mechanophore systems, multichannel visualization was achieved by blending **NPBr** and **NPNR_2_** in controlled molar ratios. Specifically, we fabricated composite samples with **NPBr**:**NPNR_2_** ratios of 3:1 (**NP31**) and 5:2 (**NP52**). These mixtures enabled sequential color activation: under low force (∼60 kg s^−2^ cm^−2^), only **NPNR_2_** activated (purple), while higher forces (∼106 kg s^−2^ cm^−2^) activate both mechanophores, producing a reddish‐shifted composite color due to the combination of purple and yellow. This differentiated activation behavior enables clear identification of the activation thresholds of each mechanophore within a single crosslinked polymer matrix, providing a basis for encoding different levels of mechanical stress into distinct optical signals.

To enable simultaneous strain mapping, optically trackable silver‐coated microbeads [61] were embedded in the PDMS matrix (Figure 1a,b). These beads act as fiducial markers for PTV, allowing for localized strain mapping during mechanical activation without interfering with the mechanochromic response. This modular system–combining mechanophores with discrete activation thresholds and microbeads for strain tracking–establishes a fully visual, real‐time sensing platform capable of reporting both stress and strain from optical signals via FUS‐controlled color transitions (Figure 1c).

### 2.2 FUS System for Tunable Mechanochromism

**FIGURE 3:**
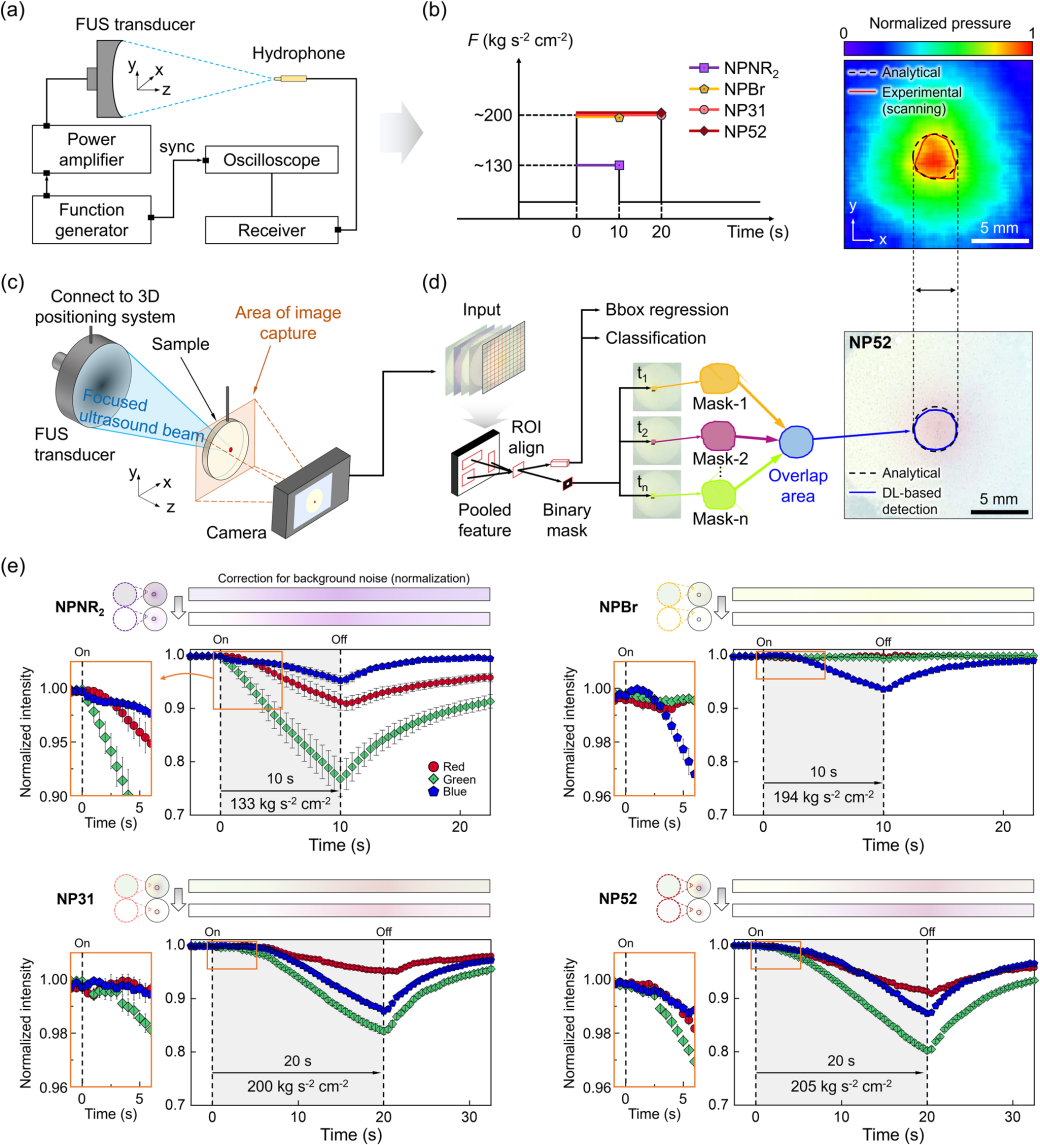
Quantification of RGB responses of mechanophore‐embedded PDMS samples using FUS: (a) Acoustic pressure measurement at the focal spot using a needle hydrophone, used to calibrate the spatial profile of the applied force distribution; (b) Applied acoustic radiation force (*F*, converted from I_SPTA_), sonication duration for each sample, and the spatial profile of the acoustic pressure (force); (c) Experimental setup for applying spatiotemporally resolved ultrasound force and capturing real‐time color changes using a waterproof digital camera; (d) Image‐based RGB quantification using a trained Mask R‐CNN deep‐learning model to segment mechanochromic regions and extract their RGB intensity profiles; and (e) Time‐resolved RGB intensity traces for each sample, normalized to a non‐responsive background region. Error bars indicate the standard deviation from three independent replicate measurements performed on a single sample for each formulation (**NPNR_2_**, **NPBr**, **NP31**, and **NP52**). Distinct color‐channel dynamics reflect mechanophore‐specific activation thresholds and temporal response profiles during FUS exposure. The full RGB intensity profiles and representative optical images, including the recovery process after FUS exposure, are provided in Figures S5 and S6.

To achieve localized and controllable mechanophore activation, we developed a FUS system capable of delivering mechanical force with high spatial and temporal precision. This system enabled non‐contact and selective activation of mechanophores embedded in PDMS by targeting specific regions, with spatial confinement governed by the ultrasound beamwidth. The FUS setup (Figure 3a) consisted of a single‐element ultrasound transducer (center frequency: ∼438 kHz; beamwidth: ∼2.9 mm; and depth‐of‐field: ∼18.3 mm), operated by a function generator (EofE Ultrasonics, South Korea) and power amplifier (EofE Ultrasonics, South Korea). The fabricated PDMS samples were immersed in a degassed water tank and acoustically coupled via a thin membrane. The acoustic focal spot (red‐colored area in Figure 3b) was aligned with the target region of each sample. The system operated in continuous wave (CW) mode, and ultrasound intensity was quantified as the spatial‐peak temporal‐average intensity (I_SPTA_) using a calibrated needle hydrophone (NH2000, Precision Acoustics, UK). The estimated I_SPTA_ ranged from 868 to 2877 W cm^−2^, depending on sample type, and was converted to the corresponding acoustic radiation force (*F*) after correcting for acoustic impedance mismatch, reflection, and transmission coefficients at the water‐PDMS interface (Tables S1 and S2). The resulting radiation force defined activation windows specific to each mechanophore formulation: approximately 61–133 kg s^−^^2^ cm^−^^2^ for **NPNR_2_**; 79–194 kg s^−^^2^ cm^−^^2^ for **NPBr**; 60–200 kg s^−^^2^ cm^−^^2^ for **NP31**; and 62–205 kg s^−^^2^ cm^−^^2^ for **NP52**. These calibrated forces were responsible for triggering color changes in the focal spot, acting as spatiotemporally confined mechanical loads (Table S2).

To rule out thermal activation, a thermocouple was placed at the focal spot. Even at the maximum force (e.g., 205 kg s^−^^2^ cm^−^^2^, corresponding to an I_SPTA_ of 2,877 W cm^−2^ for **NP52**), the temperature increased only up to ∼43°C, which is insufficient to thermally activate the mechanophores (Figures S2–S15) [54]. However, when the radiation force exceeded the maximum activation threshold for each sample, irreversible opacification was observed at the focal spot, indicating the onset of thermal ablation. All quantitative analyses were thus conducted below these thresholds to ensure fully reversible mechanochromic activation. Under these calibrated conditions, all PDMS samples exhibited repeatable color changes that fully reversed upon cessation of sonication (Figures S5 and S6). For instance, **NPBr** transitioned from a colorless state (Initial RGB: red = 227; green = 231; and blue = 195) to yellow (red = 228; green = 233; and blue = 207) under FUS and returned to its initial values within 18 s after unloading (Figures S5 and S6). Repeated stimulation at identical conditions resulted in less than 1% variation in color intensities over three cycles (Figure S5), confirming the excellent repeatability of the mechanochromic response. The duration of FUS exposure was set to 10 s for the **NPNR_2_** and **NPBr** samples, and extended to 20 s for **NP31** and **NP52** samples (see Figure 3b). The entire mechanochromic process was recorded using a waterproof digital camera to capture the spatiotemporal evolution of color changes (Movies S1–S4).

By modulating the transducer's input voltage and exposure duration, we achieved tunable control over the radiation force for each formulation. As seen in Figure 2b, **NPNR_2_**‐PDMS began to exhibit a purple color at ∼61 kg s^−^^2^ cm^−^^2^, with purple intensity increasing monotonically up to ∼133 kg s^−^^2^ cm^−^^2^ without causing any structural damage in the matrix. Similarly, **NPBr**‐PDMS produced a yellow color between ∼79 and 194 kg s^−^^2^ cm^−^^2^, confirming that **NPBr** required a higher activation threshold. The difference in initial activation threshold is in good agreement with CoGEF‐based density functional predictions, which confirms that the activation barrier for **NPNR_2_** is lower than that of **NPBr** (Figure S1b). Importantly, at higher forces (e.g., ∼200 kg s^−^^2^ cm^−^^2^), where both mechanophores are activated, composite samples **NP31** and **NP52** displayed a red‐shifted color output, as the optical combination of purple (**NPNR_2_**) and yellow (**NPBr**) mechanochromic outputs. **NP52** exhibited a deeper red than **NP31** under similar loading conditions, attributed to its higher **NPNR_2_** content. These observations indicate that the color output of dual‐mechanophore systems can be finely tuned by adjusting the mechanophore composition (Figure 2b), thereby supporting their suitability for graded and multichannel quantitative mechanochromic sensing.

### 2.3 Automated Detection of Color‐Change Area Using Deep Learning

Accurate stress quantification in the mechanophore‐embedded PDMS system requires precise identification of the localized regions activated by acoustic radiation force. To achieve this, two critical factors must be determined: (1) the accurate identification of the area where color change occurs; and (2) the quantification of mechanical radiation force delivered to that area. Given that the applied force can be derived from the conversion of I_SPTA_ to *F*, delineating the color‐change area is key for calculating local stress (= force/area) with precision. In addition, manual extraction is impractical for real‐time applications and may introduce potential user bias, requiring a robust and autonomous segmentation strategy. To fulfil this need, we developed a deep‐learning‐based image segmentation method using a Mask R‐CNN model (Figure 3d). Training and validation were performed using datasets derived from experimentally acquired time‐lapse images, undergoing more than 1600 training cycles (see Experimental Section). Given the collected images from the recorded videos, the model was trained to automatically detect and contour regions exhibiting different colors in PDMS samples subjected to *F*. The final model achieved a segmentation accuracy exceeding 97%, as confirmed by independent test sets (Figure S3). For instance, in the **NP52** sample, the extracted color‐change area obtained from the Mask R‐CNN segmentation (blue dashed line) was 6.28 mm^2^, while the areas calculated by the full width at half maximum (FWHM) beamwidth (black dashed line) (Figure S7a) and the scanned acoustic pressure field (red line) were 6.05 and 6.90 mm^2^, respectively (Figure 3d). The difference between the analytically estimated area (via FWHM) and the area obtained from the Mask R‐CNN was minimal (∼3.6%), indicating good agreement between the analytical calculation and automated segmentation. This agreement demonstrates that the Mask R‐CNN model can accurately delineate mechanically activated regions by accounting for both radial force distribution and gradual chromogenic attenuation, beyond the assumptions of simple geometric estimation by the beamwidth. Mask R‐CNN also simultaneously extracted the RGB profiles, allowing for dynamic monitoring of color changes. We confirmed that color changes and RGB profile variations were confined predominantly to the activated regions, with no significant color shift outside the focal spot. This supports that the detected areas correspond accurately to the regions subjected to localized mechanical loading. The automated and spatially precise contouring enabled by Mask R‐CNN provides a foundation for subsequent quantitative stress mapping based on mechanochromic optical signals.

### 2.4 Multichannel RGB Analysis of Time‐Dependent Mechanophore Activation

We analyzed RGB profiles within the color‐change areas extracted by the Mask R‐CNN model to track the intensity shift of each channel (Figures 3e and S4). To account for variability in imaging conditions (e.g., camera angle, surface reflectivity, ambient scattering), all RGB values were normalized to a reference region where no color change was detected (Figures 3e, S6, S12 and S14). For **NPNR_2_** (initial RGB: red = 181; green = 172; and blue = 185), a purple chromatic shift was characterized by a significant decrease in the green channel (∼23%, 172 → 132), along with smaller reductions in the red (∼9%, 181 → 165) and blue (∼5%, 185 → 176) channels. In contrast, **NPBr** (initial RGB: red = 228; green = 233; and blue = 207), which activates at higher stress thresholds, exhibited a yellow shift primarily driven by a ∼7% decrease in the blue channel, while red and green values remained almost stable (only 0.6%–0.8% decrease). For dual‐mechanophore samples **NP31** and **NP52**, the RGB profiles reflected the combined features of **NPNR_2_** and **NPBr**, resulting in reddish color tones. **NP31** (initial RGB: red = 243; green = 244; and blue = 226) showed reductions of ∼5% (243 → 231) in red, ∼16% (244 → 205) in green, and ∼12% (226 → 199) in blue, whereas **NP52** (initial RGB: red = 212; green = 213; and blue = 202), which contains 14.3% more **NPNR_2_**, exhibited greater reductions of ∼9% (212 → 193) in red, ∼20% (213 → 171) in green, and ∼13% (202 → 176) in blue, yielding a deeper red appearance.

Temporal trends in RGB profiles further elucidated the response rates and activation dynamics of mechanophores. For instance, during the first 3 s of FUS exposure, the normalized green channel in **NP52** decreased by ∼3% (1.00 → 0.97), while the blue channel dropped by only ∼1% (1.00 → 0.99), indicating earlier activation of **NPNR_2_** compared to **NPBr**, as evidenced by the appearance of a purple color. Upon cessation of FUS, the green channel of **NPNR_2_** exhibited a markedly steeper recovery slope than the blue channel of **NPBr**, indicating that **NPNR_2_** merocyanine reverts more rapidly and thus reaches its steady‐state coloration faster (Figure 3e). Note that direct comparison of the recovery times is not considered in this study, as the total magnitude of channel change differs between mechanophores. These observations are consistent with CoGEF‐based density functional calculations, which predict earlier activation of **NPNR_2_** due to its lower activation threshold (see Section 2.2 and Figure S1).

**FIGURE 4:**
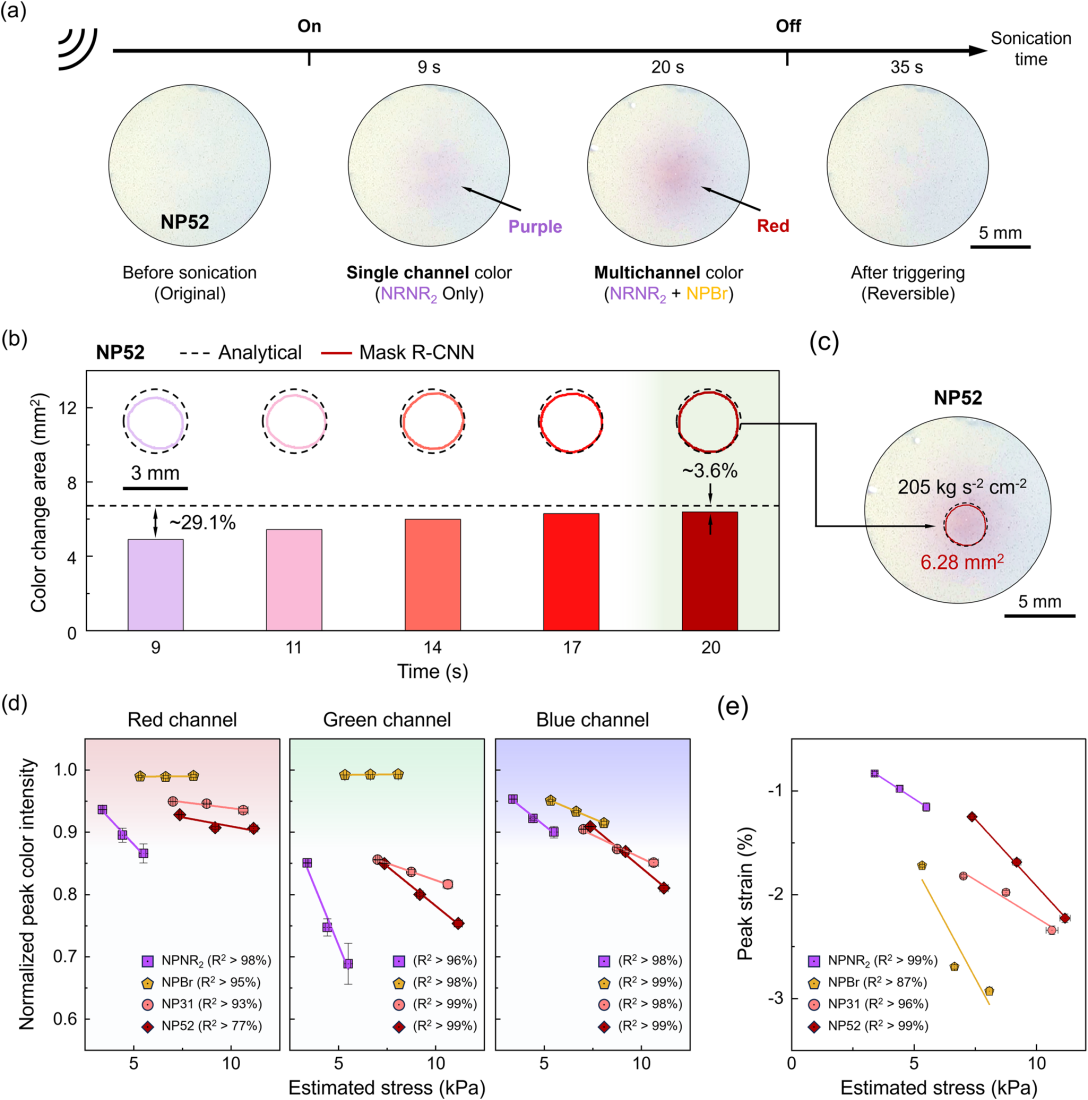
Quantification of mechanical stress in mechanophore‐embedded PDMS samples under FUS and its correlation with RGB intensity and strain: (a) Digital images showing multichannel color changes in **NP52** under FUS activation and subsequent relaxation. **NP52** yields a time‐dependent multicolor shift from purple to red, demonstrating kinetic difference between two mechanophores. All color changes are reversible upon cessation of force; (b) Temporal evolution of the color‐change area in **NP52** extracted by Mask R‐CNN. At 20s of sonication, the extracted area closely matches the analytically calculated area based on the FWHM beamwidth, with only ∼3.6% deviation (> 96% precision); (c) Example of the estimated stress for **NP52**; (d) Linear correlation between peak RGB intensities and estimated stress across all samples, confirming the chromogenic response to stress; and (e) Linear correlation between estimated peak stress and strain, confirming linear elastic behaviors of all samples. Error bars in (d) and (e) indicate the standard deviation of three independent replicates.

As shown in Figure 4a, under 205 kg s^−^^2^ cm^−^^2^ of applied force, **NP52** initially exhibited a purple color for ∼8 s (red = 206; green = 202; and blue = 198), followed by the emergence of yellow, resulting in a red appearance (red = 193; green = 171; and blue = 176) by the end of sonication (∼20 s). This progressive and mechanophore‐specific color change demonstrates the capability of **NP52** to encode different levels of mechanical input into distinct optical responses, supporting its use for multichannel quantitative mechanochromic sensing.

### 2.5 Stress Quantification Using RGB Values in Extracted Colored Area

A key consideration in stress quantification is the time‐dependent evolution of the color‐change areas upon subjecting the mechanophores to radiation force. For **NP52**, the area extracted via RGB masking (using Mask R‐CNN segmentation) showed a substantial deviation from the analytically calculated region during the early phase of sonication (Figure 4b). As the mechanochromic response developed during FUS exposure, the color‐change area gradually increased. Since the acoustic radiation force was spatially confined within the FUS beamwidth, the color‐change area eventually reached the beamwidth, reducing the deviation from ∼30% to ∼3.6% at 20 s. These transient characteristics are likely associated with the viscoelastic behavior of the PDMS matrix, which varies among formulations. The convergence of area over time is essential for ensuring accurate and reliable stress estimation. Upon this confirmation, the estimations of area were taken at time points corresponding to peak RGB intensity: 10 s for **NPNR_2_** and **NPBr**; and 20 s for **NP31** and **NP52** (Figure S10). Having established all the required information (Figure 3), including spatiotemporally calculated colored area by Mask R‐CNN, controlled radiation force, and RGB profiles, we determined the local mechanical stress induced within the colored area by dividing the estimated radiation force by the extracted color‐change area (Figure 4c). As a result, the estimated stress values are: 3.39–5.50 kPa for **NPNR_2_**, 5.33–8.07 kPa for **NPBr**, 7.02–10.63 kPa for **NP31**, and 7.36–11.16 kPa for **NP52**. Note that these values correspond to a 2D plane 0.5 mm beneath the sample surface facing the transducer, aligning with the measurement regions used for strain and RGB data. Although the color intensity was recorded from the opposite side, as described in Figure 3c, the spatial alignment of the measurement planes ensures that RGB, strain, and stress data remain physically correlated, thereby mimicking practical application scenarios where visual sensing must occur from the accessible surface.

We further analyzed the relationship between the estimated stress and peak RGB intensities for all samples (Figures 4d and S11). Although the mechanochromic response evolves during FUS exposure, the peak RGB values were obtained when the color‐change area matched the FUS beamwidth, providing a consistent criterion for stress quantification under different FUS conditions. All channels showed strong linear correlations with stress for all mechanophore types, despite the limited number of investigated stress levels. The error bars remain substantially smaller than the differences between the mean values, supporting the observed trends. Compared to the single‐mechanophore samples (**NPNR_2_** and **NPBr**, *R^2^* > 95%), the composite samples (**NP31** and **NP52**) exhibited intermediate linear slopes (77% < *R^2^* < 99%), indicating simultaneous activation of both mechanophores. **NP52**, which contains a higher fraction of **NPNR_2_**, showed a slope more closely aligned with that of **NPNR_2_**. **NP52** showed a gradual color transition from purple to red, which became more evident with increasing stress. Notably, the normalized red peak intensity for **NP52** initially dropped sharply (0.93 → 0.90) at low stress (7.36 kPa → 9.18 kPa) and then remained relatively stable (0.90 → 0.90) at higher stress levels (9.18 kPa → 11.16 kPa). The nonlinear behavior observed in the red channel for **NP52** at 20 s is attributed to the significant effect of **NPNR_2_** at low stress levels, followed by **NPBr** activation at high stress. Since **NP52** shows a linear stress‐strain relationship (*R* ^2^> 99%) over the measured range (Figure 4e), the effect of the nonlinear elastic response of the PDMS matrix is likely to be minor. In contrast, **NP31**, despite containing both mechanophores, did not exhibit a similar nonlinear trend in the red channel, indicating that the ratio of mechanophores is a critical factor for tuning multichannel mechanochromism. Taken together, these results show that the combined RGB profile, accounting for both the linear responses of the green and blue channels and the nonlinear behavior of the red channel, can be used to quantitatively evaluate localized mechanical stress. Notably, this approach establishes mechanophore responses not only as visualization signals but also as quantitative sensing outputs within a bulk polymer system.

### 2.6 Strain Analysis Using PTV

**FIGURE 5:**
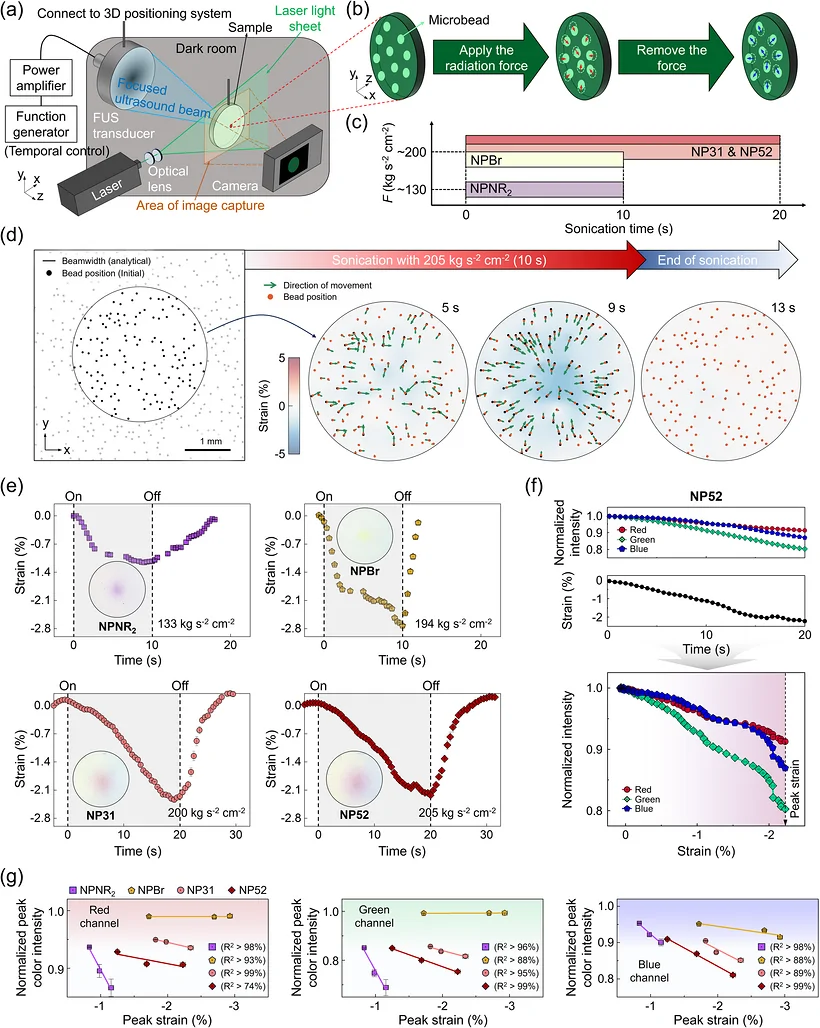
Quantification of strain and correlation with RGB response in mechanophore‐embedded PDMS samples under FUS: (a) Schematic of the strain measurement setup and PDMS sample where silver‐coated tracer beads were embedded and illuminated by a laser during FUS stimulation; (b) Time‐sequenced displacement patterns of the beads during sonication, showing radially symmetric inward motion followed by full recovery upon cessation of the radiation force; (c) Summary of the applied radiation force and FUS duration for each sample; (d) Bead motion trajectories and the corresponding estimated strain field during and after sonication, indicating radial convergence and relaxation corresponding to reversible deformation; (e) Strain‐time profiles under peak force, showing the mean strain calculated from all tracked beads within the FUS beamwidth, demonstrating mechanophore‐specific deformation and recovery dynamics; error bars denote standard deviations from three replicate measurements; (f) Time‐resolved RGB intensity and strain profiles for **NP52**, along with a strain‐RGB correlation during FUS exposure; and (g) Correlation between peak strain and peak RGB intensities for each channel across all samples (linear fit: *R* ^2^ = 74–99%).

During the application of radiation force to the mechanophore‐embedded PDMS, localized deformation was induced within the designated colored region. Previous studies [29, 49, 57, 58, 62] have linked mechanochromic color changes to force; however, direct and quantitative extraction of strain from mechanochromic optical signals remains challenging, particularly in bulk solid polymer systems, due to the directional mismatch between the strain field and chromogenic response. To address this limitation, we implemented the PTV method [61, 63] (Figure 5a), which quantifies strain by tracking the displacement of embedded tracer beads under FUS stimulation (see Experimental Section).

The radiation force was modulated across all mechanophore samples in the range of 133–205 kg s^−^^2^ cm^−^^2^, with FUS applied for 10 s for the **NPNR_2_** and **NPBr** samples and extended to 20 s for **NP31** and **NP52** (Figure 5c). As shown in Figure 5d and Movies S5–S8, the embedded beads exhibited radially symmetric inward displacements confined within the beamwidth‐defined region. Upon cessation of the force, the beads gradually returned to their initial positions, confirming fully reversible deformation behavior. The strain of each tracked bead was calculated from its radial displacement, and the resulting strain values within the FUS beamwidth were averaged to generate the time‐resolved strain profiles (Figure 5e). Peak compressive strains along the radial direction at the end of sonication were approximately −1.2% for **NPNR_2_**, −2.8% for **NPBr**, −2.3% for **NP31**, and −2.2% for **NP52**. The corresponding recovery times to near‐zero strain were ∼9, 2, 7, and 6 s, respectively. These results confirm that the FUS system can generate localized and reversible strain fields within the PDMS matrix. We observed a strong linear correlation (*R* ^2^> 87%) between peak strain and stress across all samples (Figure 4e), indicative of linear elastic deformation within the tested range. Based on these data, the elastic moduli were estimated to be ∼461 kPa for **NPNR_2_**, ∼262 kPa for **NPBr**, ∼440 kPa for **NP31**, and ∼510 kPa for **NP52**. Although all formulations are based on the same PDMS matrix, variations in the curing and crosslinking processes during sample preparation may result in differences in mechanical properties. These differences are reflected in the estimated elastic moduli and the corresponding strain recovery behaviors following FUS unloading.

Next, we examined the time‐dependent strain data (Figure 5e) with RGB profiles (Figure 3e for **NP52)** to correlate strain and RGB intensity. Although both showed similar temporal trends, RGB intensity remained nearly unchanged during the first 2–3 s of sonication while strain continued to develop, indicating a phase lag between deformation and color change. To account for these coupled dynamics, we implemented a correlation framework that captures the concurrent evolution of RGB intensities and strain during FUS exposure (Figure 5f). This analysis enables direct and quantitative extraction of strain states from optical readouts, establishing a critical link between mechanochromic response and mechanical deformation. Lastly, we analyzed the correlation between peak strain and peak RGB intensity across all samples (Figure 5g). Although the green and blue channels showed slightly reduced *R* ^2^ (> 88%) compared to the stress‐RGB correlation (*R* ^2^> 98%), they still exhibited linear relationships with strain. In contrast, the red channel in **NP52** exhibited a marked deviation from linearity (*R* ^2^ = 74%), echoing the sequential activation behavior observed in the stress‐RGB analysis. In this case, the normalized red peak intensity decreased from 0.93 to 0.90 at low strain levels (−1.2% to −1.7%), then remained nearly unchanged (0.90 to 0.90) as strain increased further (−1.7% to −2.2%). Similar to the stress analysis, this nonlinear trend arises from the sequential chromogenic response of the **NPNR_2_** and **NPBr** mechanophores, where **NPBr** activation follows that of **NPNR_2_** at higher force levels.

Although the strain values were obtained from tracer‐bead displacement rather than directly from mechanophore reactions, the correlation still provides useful insight into how macroscopic deformation translates into molecular‐level activation. These observations demonstrate that strain‐induced chromogenic outputs can be quantitatively interpreted through RGB analysis, providing a robust optical readout of local mechanical deformation. The overall trend further indicates that the chromogenic response is strain‐correlated, likely reflecting strain‐mediated stress transfer to the mechanophore moieties. These results demonstrate that optical signals can be used to quantitatively estimate strain in a bulk polymer system, enabling optical sensing of both stress and strain selectively from mechanochromic responses.

### 2.7 RGB‐Stress–Strain Correlation and Potential Applications

**FIGURE 6:**
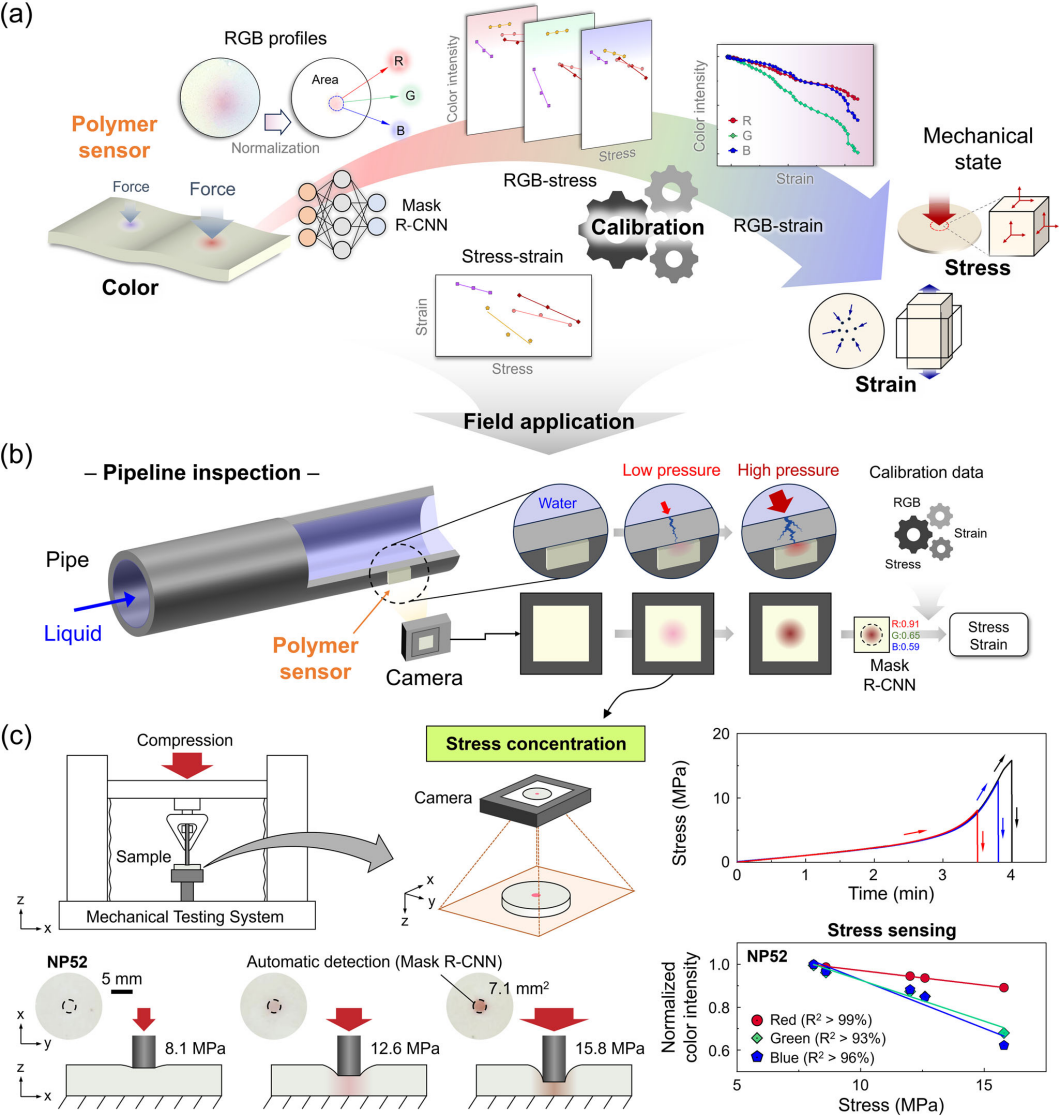
RGB‐stress–strain correlation and potential applications of mechanophore‐embedded PDMS sensors. (a) Schematic illustration of the mechanochromic sensing framework enabling selective estimation of stress or strain from colorimetric changes. (b) Conceptual application scenario: polymer sensors attached to pressurized pipelines non‐invasively visualize localized stress development, with color outputs rapidly analyzed through pre‐established RGB‐stress‐strain profiles. (c) Proof‐of‐concept validation under uniaxial compression: the **NP52** sample was subjected to monotonically increasing mechanical loading (8.1‐15.8 MPa) using an MTS. Color changes were segmented and analyzed to yield RGB‐stress curves, showing strong linearity for all channels (*R* ^2^ > 93%), demonstrating the potential of RGB‐based stress sensing.

We quantitatively analyzed the changes in RGB intensity, stress, and strain within the regions of mechanophore‐embedded PDMS activated by acoustic radiation force. In the previous sections, we established comprehensive correlations among RGB‐stress, RGB‐strain, and stress–strain relationships of the material. It is noteworthy that, although the applied stress remains constant after the onset of ultrasound exposure, the strain continues to increase over 10–20 s, mainly due to the inherent viscoelastic nature of polymers. This phase lag implies that stress can be accurately estimated only after strain has sufficiently developed. As discussed in Section 2.6, another phase lag was observed between strain and RGB change. These time‐dependent responses indicate that the current sensing platform is best suited for quasi‐static or slowly varying loading conditions, where mechanical changes typically evolve over extended durations, as commonly observed in large‐scale infrastructure including bridges and dams. For transient loading, rapid impact, or short‐duration dynamic monitoring, further characterization of the time‐dependent RGB‐strain–stress response under different loading rates would be required. Within this quasi‐static operating regime, this mechanochromic sensing strategy (Figure 6a) demonstrates a new opportunity to harness mechanophores for non‐invasive monitoring of mechanical deformation in critical infrastructure components. One possible application is the nondestructive evaluation (NDE) of steel pipelines used in oil and gas transport networks (Figure 6b). These systems are exposed to complex loading conditions that can lead to localized stress concentration and strain accumulation over time. Under typical operational conditions, steel pipelines experience strain values in the range of 0%–0.2%, with values between 0.2% and 0.5% approaching the yield threshold [64]. When strain exceeds 0.5%, plastic deformation or structural failure becomes the onset. As depicted in Figure 6a,b, our mechanophore‐embedded PDMS sensor is capable of capturing localized stress and strain within these critical regimes. A key advantage of this sensor lies in its ability to quantify both stress and strain from a single visual output when desired–a capability not accessible with existing optical or conventional mechanical sensing approaches.

As a proof‐of‐concept demonstration of the RGB‐based stress sensing strategy under realistic conditions, the **NP52** sample was subjected to uniaxial compression to evaluate its stress‐sensing capability (Figure 6c). Controlled compressive loads, mimicking internal hydraulic pressure, were applied using a conventional mechanical testing system (MTS). Although this loading was designed to mimic internal hydraulic pressure, discrepancies in stress and strain are expected because the boundary conditions and stress/strain distributions differ from those in an actual steel pipeline. Different sets of monotonically increasing forces were applied to the **NP52**‐PDMS sample, each held for at least 3 min to obtain different steady‐state RGB values. The RGB response was confirmed to reach saturation within this loading period (Figure S16). Due to spatial constraints in the loading setup, strain measurements were not performed in this experiment. Compared to the instantaneous loading (only 20 s) from the FUS system, this prolonged loading period was long enough to obscure the multichannel color response (purple to red) induced by the different activation thresholds of the mechanophores. As a result, only a red‐phase signal was captured by the imaging system. As the applied stress increased from 8.1 to 15.8 MPa, a monotonic increase in red coloration was observed within the stressed regions. The color‐change areas were segmented using a Mask R‐CNN model, followed by RGB intensity analysis, which revealed strong linear correlations with the applied stress across all channels (*R* ^2^> 93%), supporting the feasibility of the proposed RGB‐based stress sensing strategy (Figure 6c). These results demonstrate that the mechanochromic sensor enables spatially resolved and quantitatively reliable visualization of stress distributions under mechanical compression. Note that the MTS result should be interpreted independently from the FUS‐based stress quantification at this stage, as the two methods differ in loading type, boundary conditions, stress distribution, and time scale. Future work will investigate how loading conditions affect the RGB‐stress relationship and extend the calibration framework under different loading modes and boundary conditions.

Beyond their quantitative performance, these mechanochromic sensors offer potential advantages for practical deployment. Their thin and flexible structure allows conformal attachment to complex geometries, including potential applications such as pipe joints, valve housings, and bearing surfaces, enabling visualization of localized stress evolution. The use of optical readout eliminates the need for embedded electronics, thereby reducing deployment constraints in environments where conventional electronic sensors are vulnerable to damage or failure. Moreover, their reversible mechanochromic response permits repeated measurements under cyclic loading without signal degradation. Further studies are nonetheless required to evaluate sensor durability, environmental stability, response kinetics under different loading durations and rates, and the associated changes in sensing performance under long‐term cycling. In conclusion, these results establish mechanophore‐embedded polymers as a scalable platform for quantitative sensing, enabling spatially resolved measurement of both stress and strain in complex engineering systems.

## 3 Conclusions

In this study, we developed a multichannel mechanochromic sensing platform that integrates two mechanophores within a PDMS matrix, enabling quantitative extraction of localized stress and strain through spatiotemporally controlled ultrasound force. By tuning the chemical composition of the mechanophores (**NPBr** and **NPNR_2_**) with distinct activation thresholds, we achieved sequential, force‐specific color changes that transform molecular‐level mechanochemical events into macroscopically detectable optical outputs. Unlike conventional methods that rely on global loading and qualitative interpretation, our system leveraged precise FUS for localized force delivery and integrated multiple analysis tools, including Mask R‐CNN for automated color segmentation and PTV for strain quantification, to establish strong correlations between RGB signals and mechanical parameters. These results demonstrate that mechanochromic responses provide not only visual indication, but also quantitative sensing outputs for both stress and strain in a single bulk polymer system. To our knowledge, this is the first mechanophore‐based bulk polymer platform capable of quantitatively extracting localized stress or strain from mechanochromic signals. As a structural sensing platform, its key advantage lies in providing quantitative mechanical readouts from a single visual signal without embedded electronics. Combined with the inherent flexibility and scalability of polymer matrices, this sensing approach makes the system well suited for deployment in NDE and structural health monitoring scenarios, particularly in inaccessible, submerged, or harsh environments. Furthermore, the polymer‐based design is amenable to transformation into various forms such as patches, wires, or bulk components, depending on the target structure and monitoring environment. Overall, this proof‐of‐concept work demonstrates how mechanochromic materials can evolve from laboratory prototypes into practical diagnostic tools. By bridging molecular mechanics with data‐driven analysis and programmable force actuation, this work establishes a pathway toward non‐invasive, polymer‐based mechanical sensing for next‐generation infrastructure and safety systems.

## 4 Experimental Section

### 4.1 Fabrication of PDMS Mixtures with Mechanophores

Mechanophore‐embedded PDMS samples were fabricated by blending PDMS oligomer, silver‐coated microbeads with an average diameter of ∼40 µm, and a curing agent at defined mass ratio, followed by solvent evaporation, degassing, and a thermal curing. Specifically, the mechanophores (either **NPBr** or **NPNR_2_**) were incorporated at 1.5 wt.%, the silver‐coated microbeads at 0.015 wt.%, and the curing agent at 0.1 wt.%, relative to the total PDMS oligomer mass. To ensure uniform dispersion of the solid components, the mixture, excluding the curing agent, was first dissolved in xylene and stirred thoroughly at room temperature. The resulting suspension was then placed on a hot plate at a mild temperature to allow gradual evaporation of the xylene solvent. Once the solvent was fully removed, the curing agent was added to initiate crosslinking, and the mixture was subjected to vacuum degassing in a desiccator to eliminate entrapped air bubbles. The degassed mixture was poured into cylindrical molds and cured at 80°C overnight on a hot plate to complete the crosslinking process. The final cured samples were elastomeric disks with a uniform thickness of ∼5.0 mm and a diameter of ∼5 cm. These dimensions were selected to facilitate uniform FUS exposure during subsequent mechanochromic and strain‐mapping experiments. Microscopic observation of the prepared PDMS composites confirms that the microbeads are uniformly distributed throughout the matrix (Figure S13). In this study, a single sample was fabricated and tested for each formulation (**NPNR_2_**, **NPBr**, **NP31**, and **NP52**). All samples were stored in a dark, dry environment to prevent premature photochemical activation of the mechanophores.

### 4.2 FUS Beam Characterization and Acoustic Radiation Force

The spatial distribution and focal precision of the FUS beam were characterized by mapping the pressure field in a degassed water tank using a 2.0 mm needle hydrophone (NH2000, Precision Acoustics, UK). The FUS transducer used in this study was spherically focused, with a nominal center frequency of 438 kHz and a focal length of 45 mm (*f*‐number is 0.9). Beam profiling was conducted in both the lateral (x‐y) and axial (z) directions to evaluate the FWHM of the pressure field. Under standard operating conditions, the lateral beamwidth and axial depth of field were measured to be approximately 2.96 mm and 18.33 mm, respectively. These values indicate strong spatial confinement of acoustic energy, suitable for localized radiation force application. The hydrophone voltage signals were converted to acoustic pressure using the sensitivity of ∼4.6 V MPa^−1^ at 438 kHz (Figure S9). The value of I_SPTA_ was calculated from the peak acoustic pressure amplitude (*p*) using the equation:

(1) $$I_{\mathrm{SPTA}}=p^{2}{\left(2\rho c\right)}^{-1}$$

where ρ is the density of the propagation medium and *c* is the speed of sound. For PDMS‐like materials, we used ρ = 870 kg m^−3^ and *c* = 1,170 m s^−1^. The calculated I_SPTA_ ranged from 868 to 2877 W cm^−2^ depending on the transducer voltage input and the focal distance (Table S2). Reflection losses at the water‐PDMS interface were considered in the intensity adjustment. A reflection coefficient of approximately −0.186 was used, corresponding to a ∼3.4% reduction in transmitted acoustic power. The acoustic radiation force per unit volume (*F*) applied to the PDMS disk was computed from the intensity using the following relationship:

(2) $$F=2\alpha I_{\mathrm{SPTA}}c^{-1}$$

where α is the absorption coefficient (in dB m^−1^) (Table S1), and *F* is expressed in units of kg s^−2^ m^−2^ (Table S2). Given that attenuation in PDMS is primarily due to absorption, with scattering effects being negligible, the measured α was directly used to calculate the acoustic radiation force. This computed force was used in subsequent analyses to correlate acoustic input with mechanophore activation and local stress estimation.

### 4.3 Spatiotemporal Control of Mechanochromic Response

To confirm that the observed mechanochromic responses originated from mechanical stimulation rather than thermal effects, the local temperature at the focal spot was monitored using a thermocouple during ultrasound exposure. At the maximum tested I_SPTA_ (2877 W cm^−^^2^) applied for 20 s, the temperature increased up to ∼42.5°C, an approximately 14°C rise relative to the baseline temperature of 28.8°C (Figure S2). This temperature remained well below the threshold required to thermally activate the embedded NP derivatives, confirming that color changes were mechanically driven (Figure S15). Temporal control of ultrasound delivery was achieved by precisely adjusting key sonication parameters, including pulse repetition rate, pulse length, number of cycles, and duty cycle (illustrated in Figure S7b). These settings were controlled using a power amplifier and function generator (Figure 3a), enabling reproducible stimulation sequences for systematic studies across multiple samples. For spatial control, a three‐axis motorized positioning system was employed to place the focal spot of the FUS transducer within ∼10 µm of the PDMS film surface. The focal distance between the transducer and the sample surface was precisely maintained to ensure consistent energy delivery to the target region. Throughout the FUS application, the entire mechanochromic activation process was recorded in real time using a waterproof digital camera placed beneath the tank. These recordings enabled dynamic analysis of RGB color evolution and subsequent correlation with localized stress and strain fields.

### 4.4 PTV Analysis for Strain Calculation

For accurate quantification of strain, PTV was employed using silver‐coated microbeads as fiducial markers embedded within the PDMS matrix (0.015 wt.%). These particles, with an average diameter of ∼40 µm, were chosen for their scale relative to the laser wavelength (approximately 532 nm), which allows omnidirectional light scattering and enhances optical visibility under laser illumination (Figure 5a). A laser sheet was generated by a double‐pulsed Quantel laser (250 mJ/pulse) to illuminate the tracer beads within the focal region of the FUS field. An 8 MP CCD PowerView camera (3320 × 2496 pixels) equipped with a Nikon AF Micro‐Nikkor lens (focal length of 105 mm and f/2.8 focal ratio) was used to record the bead motion at 25 Hz in high‐resolution time‐lapse image pairs before, during, and after the FUS exposure (Movies S5–S8). The optical setup ensured that bead displacements were clearly visible even under low‐intensity scattering conditions. As illustrated in Figure S8, a multi‐step preprocessing pipeline was implemented to enhance particle recognition accuracy and improve the signal‐to‐noise ratio (SNR). First, the region of interest (ROI) from the original RGB image was cropped and split into its green channel, which exhibited the highest bead contrast. This green channel was then converted to grayscale to suppress color‐induced variations and emphasize intensity differences associated with bead positions. Next, a two‐stage filtering method was applied to isolate bead contours and minimize background noise. A local mean filter was used to smooth out high‐frequency variations while preserving particle shape, followed by a Gaussian blur to further stabilize particle boundaries and reduce artifacts. This filtering process markedly improved image clarity, as shown in Figure S8b, enabling more accurate binary conversion and centroid detection. Following preprocessing, the grayscale images were binarized to extract bead locations. The centroid coordinates of the binarized particles were identified and used as input for tracking algorithms that mapped their displacement across sequential frames. Particular care was taken to avoid misinterpretation of bead clusters as single particles, which could lead to underestimation of local strain. The processed particle trajectories were then used to compute local strain fields [65, 66]. Specifically, the displacement vector of each tracked bead was projected onto the radial direction defined from the bead's initial position toward the FUS focal center. The strain (ε) was calculated by normalizing the projected radial displacement by the bead's initial radial distance:

(3) $$\varepsilon =\frac{u_{r}}{r_{0}}$$

where *u_r_* is the projected radial displacement and *r*_0_ is the initial radial distance between the bead and the FUS focal center. The strain values obtained at individual bead locations were spatially interpolated to generate continuous strain maps. The resulting strain maps provided spatially resolved mechanical response data, enabling direct correlation with the mechanochromic activation patterns.

### 4.5 Deep Learning Model Architecture and RGB Data Extraction

To extract spatially resolved RGB profiles from mechanochromic PDMS films and enable quantitative mapping of stress, a deep learning–based image segmentation approach was implemented. The model was designed to operate reliably across variable experimental conditions, including changes in lighting intensity, background texture, and mechanophore composition. To ensure this robustness, a diverse training dataset was curated, comprising over 1600 manually annotated image instances encompassing a wide range of imaging conditions. The segmentation framework was built upon the Mask R‐CNN architecture, employing a ResNet‐50 backbone pretrained on the COCO dataset. Model training was conducted using a learning rate of 0.00025, a batch size of 2, and a total of 2500 iterations (Figure S3). This configuration enabled accurate detection of mechanochromic activation zones and reliable extraction of red, green, and blue (RGB) intensity values from the identified regions. The trained model achieved an overall segmentation accuracy exceeding 97%, as validated against a reserved test set.

Extracted RGB data from localized color‐change regions were normalized against reference background values and used to generate pixel‐level intensity maps. These data formed the basis for a correlation analysis, linking RGB profiles to independently measured local stress and strain values. Using this RGB–mechanics mapping, the model enabled reverse estimation of stress magnitudes directly from colorimetric outputs, without the need for embedded sensors or strain gauges. This automated analysis pipeline was central to quantifying mechanical input in mechanochromic PDMS films and establishing a visual force‐sensing modality. All RGB values used in stress estimation were obtained from the focal region of the PDMS film, corresponding to the area of peak acoustic force application.

### 4.6 Acoustic and Physical Properties of Mechanophore‐embedded PDMS Samples

The acoustic behavior of the mechanophore‐embedded PDMS samples was characterized using time‐of‐flight (TOF) and attenuation measurements. These measurements were conducted with a planar single‐element ultrasound transducer operating at a center frequency of 438 kHz in pulse‐echo mode. A pulser/receiver system (JSR DPR300) with a 1 kHz pulse repetition frequency was employed. The transducer was aligned perpendicularly to the PDMS sample surface and immersed in a water tank maintained at 23 ± 2°C, with a fixed source‐to‐sample distance of 5 cm. Reflected echoes from the front and back surfaces of a 5 mm‐thick PDMS sample were acquired and averaged over 256 cycles to enhance SNR. The speed of sound (*c*) was determined using the TOF between the reflection peaks, yielding an average value of 1059 ± 4.2 m s^−^^1^. This result is consistent with standard PDMS materials, indicating that the incorporation of mechanophores does not significantly alter acoustic propagation properties. The measured sample density was approximately 1.105 ± 0.06 g cm^−^^3^. From this, the acoustic impedance (Z_0_ = *ρc*) ranged from 1.107 to 1.242 MRayl across different formulations. Attenuation was evaluated using a pulse‐echo insertion loss technique [67], and the attenuation coefficient (*α*) at 438 kHz was calculated as:

(4) $$\alpha \left(f\right)=10{\left(2d\right)}^{-1}\log_{10}\left(P_{r}\left(f\right)P_{s}{\left(f\right)}^{-1}\right)$$

where *d* is the sample thickness, and *P_r_* (*f*) and *P_s_* (*f*) are the power spectra of the reflected waveforms with and without the PDMS sample, respectively. The average attenuation coefficient was approximately 1.32 ± 0.09 dB cm^−1^ at 438 kHz, confirming the low‐loss acoustic nature of the PDMS medium. A complete summary of the acoustic and physical properties of all tested formulations is provided in Table S1.

## Author Contributions

**Seungo Baek**: methodology, Writing – original draft, investigation, validation, formal analysis, data curation. **Jeong Hoon Rhee**: formal analysis, investigation, validation. **Skylar K. Osler**: writing – original draft, validation, formal analysis, data curation, investigation. **Minsoo P. Kim**: writing – original draft, investigation, validation, formal analysis. **Shyuan Cheng**: data curation, formal analysis, validation, writing – original draft. **Leonardo P. Chamorro**: writing – review and editing, validation, formal analysis, methodology. **Hyunhyub Ko**: investigation, validation, formal analysis, writing – review and editing. **Maxwell J. Robb**: methodology, supervision, writing – review and editing. **Gun Kim**: conceptualization, methodology, supervision, project administration, writing – review and editing, writing – original draft, funding acquisition.

## Conflicts of Interest

The authors declare no conflicts of interest.

## Supporting information




**Supporting File 1**: advs77607‐sup‐0001‐SuppMat.docx.

**Supporting File 2**: advs77607‐sup‐0002‐MovieS1.mp4.

**Supporting File 3**: advs77607‐sup‐0003‐MovieS2.mp4.

**Supporting File 4**: advs77607‐sup‐0004‐MovieS3.mp4.

**Supporting File 5**: advs77607‐sup‐0005‐MovieS4.mp4.

**Supporting File 6**: advs77607‐sup‐0006‐MovieS5.mp4.

**Supporting File 7**: advs77607‐sup‐0007‐MovieS6.mp4.

**Supporting File 8**: advs77607‐sup‐0008‐MovieS7.mp4.

**Supporting File 9**: advs77607‐sup‐0009‐MovieS8.mp4.

## Data Availability

The data that support the findings of this study are available from the corresponding author upon reasonable request.
