# Supplementary material for: Mechanophore‐Based Multichannel Colorimetric Force Sensors: Ultrasound‐Driven Stress and Strain Quantification
Source: Adv Sci (Weinh). 2026 Sep 8:e77607. Online ahead of print. doi: 10.1002/advs.77607 (PMC13554377; doi:10.1002/advs.77607)
Supplement: Supplementary file 1 [file ADVS-9999-e77607-s004.docx]

­­­Supporting Information

Mechanophore-Enabled Multichannel Optical Force Sensors: Ultrasound-Driven Stress and Strain Quantification

Seungo Baek^1^, Jeong Hoon Rhee^1^, Skylar K. Osler^1^, Minsoo P. Kim^1^, Shyuan Cheng, Leonardo P. Chamorro, Hyunhyub Ko, and Maxwell J. Robb*, and Gun Kim*

*^1^* They are equally contributed.

Seungo Baek, Gun Kim

School of Civil and Environmental Engineering, Nanyang Technological University, 50 Nanyang Avenue, Singapore 639798, Singapore

E-mail: [gun.kim@ntu.edu.sg](mailto:gun.kim@ntu.edu.sg)

Jeong Hoon Rhee

Department of Structural Engineering Research, Korea Institute of Civil Engineering and Building Technology, Gyeonggi-do 10233, Republic of Korea

Skylar K. Osler, Maxwell J. Robb

Division of Chemistry and Chemical Engineering, California Institute of Technology, Pasadena, CA 91125, USA

E-mail: [mrobb@caltech.edu](mailto:mrobb@caltech.edu)

Minsoo P. Kim

Department of Chemical Engineering, Sunchon National University, Suncheon 57922, Republic of Korea

Shyuan Cheng, Leonardo P. Chamorro

Department of Mechanical Science and Engineering, University of Illinois at Urbana-Champaign, Urbana, IL 61801, USA

Hyunhyub Ko

School of Energy and Chemical Engineering, Ulsan National Institute of Science and Technology (UNIST), Ulsan 44919, Republic of Korea

**This PDF file includes:**

Materials and Methods

Supplementary Tables (S1 to S2)

Supplementary Figures (S1 to S16)

Captions for Movies (S1 to S8)

References for SI reference citations

**Other supplementary materials for this manuscript include the following:**

Movies S1 to S8

**SI Materials and Methods**

**General Synthetic Details.** Reagents from commercial sources were used without further purification. Dry THF was obtained from a Pure Process Technology solvent purification system. Column chromatography was performed on a Biotage Isolera system using SiliCycle SiliaSep HP flash cartridges. NMR spectra were recorded using a 400 MHz Bruker Avance III HD with Prodigy Cryoprobe. The ^1^H NMR spectrum is reported in d units, parts per million (ppm), and is measured relative to the signal for residual acetone (2.05 ppm) in deuterated acetone. The ^13^C NMR spectrum was measured in deuterated acetone and is reported in ppm relative to the signal for acetone (206.26 ppm). Multiplicity and qualifier abbreviations are as follows: s = singlet, d = doublet, t = triplet, m = multiplet, br = broad, app = apparent. High resolution mass spectra (HRMS) were obtained via direct injection on an Agilent 1260 Infinity II Series HPLC coupled to a 6230 LC/TOF system in electrospray ionization (ESI+) mode. **NPBr-OH** and **NPNR_2_** were synthesized according to the reported literature method.^[1]^

**Synthesis of NPBr**

**5-(2-(pent-4-enoyloxy)ethoxy)-3-(4-(pent-4-enoyloxy)phenyl)-3-(4-bromophenyl)-3*H*-naphtho[2,1-*b*]pyran (NPBr).** A flame-dried 100 mL two-neck round bottom flask equipped with a stir bar was charged with 4-dimethylaminopyridine (54 mg, 0.44 mmol). The flask was evacuated and backfilled with N_2_ (3×), and a solution of **NPBr-OH** (720 mg, 1.47 mmol) in anhydrous THF (15 mL) was added via syringe. Triethylamine (0.65 mL, 4.7 mmol) was added via syringe. Pentenoic anhydride (724 mg, 3.97 mmol) in THF (5 mL) was added dropwise via syringe over 5 min, and the reaction was stirred at room temperature for 16h. The reaction was diluted with EtOAc (250 mL), washed with saturated aqueous NH_4_Cl (150 mL), saturated aqueous NaHCO_3_ (150 mL), and brine (100 mL). The organic layer was dried over Na_2_SO_4_, filtered, and concentrated. Purification by column chromatography on silica gel (3–30% EtOAc/hexanes) followed by a second chromatographic separation on silica gel (0–15% Et_2_O/CH_2_Cl_2_:hexanes (1:1)) afforded the title compound as a yellow oil (815 mg, 85%).

^1^H NMR (400 MHz, Acetone) δ: 7.99 (dd, *J* = 8.1, 1.3 Hz, 1H), 7.73 – 7.68 (m, 1H), 7.66 – 7.61 (m, 2H), 7.60 – 7.56 (m, 2H), 7.53 – 7.47 (m, 3H), 7.39 – 7.29 (m, 3H), 7.12 – 7.05 (m, 2H), 6.62 (d, *J* = 9.9 Hz, 1H), 5.90 (ddt, *J* = 16.3, 9.7, 6.2 Hz, 1H), 5.82 (ddt, *J* = 16.7, 10.3, 6.4 Hz, 1H), 5.11 (dq, *J* = 17.2, 1.7 Hz, 1H), 5.07 – 4.98 (m, 2H), 4.91 (ddd, *J* = 10.2, 2.5, 1.5 Hz, 1H), 4.61 – 4.54 (m, 2H), 4.47 – 4.39 (m, 2H), 2.65 (t, *J* = 7.3, 2H), 2.50 – 2.39 (m, 4H), 2.37 – 2.30 (m, 2H).

^13^C NMR (101 MHz, Acetone) δ: 173.1, 171.7, 151.3, 148.7, 145.2, 143.0, 142.8, 137.8, 137.7, 132.1, 130.7, 129.4, 129.2, 128.4, 128.2, 126.0, 125.6, 125.4, 122.4, 122.2, 122.1, 121.2, 116.8, 116.1, 115.9, 110.5, 82.3, 67.8, 63.4, 34.01, 33.99, 29.64, 29.56.

TLC (25% EtOAc/hexanes): R*_f_* = 0.58

HRMS (ESI m/z): calcd for [C_37_H_34_O_6_Br]^+^ (M+H)^+^, 653.1539; found 653.1522.

**Supplementary Tables**

**Table S1.** Material properties and acoustic parameters of PDMS with the NP mechanophore**.**

|  | **NPNR_2_** | **NPBr** | **NP31** | **NP52** |
| --- | --- | --- | --- | --- |
| Thickness (cm) | 0.413 | 0.415 | 0.531 | 0.544 |
| Weight (g) | 5.417 | 5.499 | 12.354 | 12.302 |
| Diameter (cm) | 4 | 4 | 5 | 5 |
| Volume (cm^3^) | 5.19 | 5.22 | 10.4 | 10.7 |
| Density (g/cm^3^) | 1.04 | 1.05 | 1.18 | 1.15 |
| Speed of sound (m/s) | 1,064 | 1,058 | 1,053 | 1,062 |
| Reflection coefficient | 0.144 | 0.142 | 0.087 | 0.096 |
| Transmission coefficient | 0.856 | 0.858 | 0.913 | 0.904 |
| Attenuation coefficient (dB/cm) | 1.274 | 1.455 | 1.353 | 1.211 |

**Table S2.** Relationship between I_SPTA_ and acoustic radiation force.

| I_SPTA_ (W/cm^2^) | Acoustic radiation force (kg s⁻^2^ cm⁻^2^) | | | |
| --- | --- | --- | --- | --- |
|  | **NPNR_2_** | **NPBr** | **NP31** | **NP52** |
| 868 | 61 | 59 | 60 | 62 |
| 1170 | 82 | 79 | 81 | 83 |
| 1525 | 107 | 103 | 106 | 109 |
| 1899 | 133 | 128 | 132 | 135 |
| 2368 | 166 | 160 | 165 | 169 |
| 2877 | 202 | 194 | 200 | 205 |

**Supplementary Figures**

**Figure S1.** (a) Photographic images of PDMS samples incorporating **NPNR_2_**, **NPBr**, and their mixture (**NP31** and **NP52**) following activation by UV irradiation and mechanical impact. Upon UV exposure, uniform color change was observed across the entire sample, indicating a lack of spatial control in activation. In contrast, mechanical activation via localized hammer impact produced spatially confined color changes, but with limited control over the applied force magnitude. (b) CoGEF ^[2]^ results comparing the mechanochemical response of **NPNR_2_** and **NPBr**. The activation barrier for **NPNR_2_** was lower than that of **NPBr**.

**Figure S2.** Temperature measurement at the focal spot during FUS exposure. Under the applied sonication conditions, the temperature of the PDMS sample increased to a maximum of 43 °C, which is insufficient to thermally activate the mechanophores or induce thermal damage to the PDMS ^[5]^.

**Figure S3.** Recognition accuracy and the losses of 2,500 iterations training process in Mask R-CNN model ^[6]^. (a) Accuracy of classifying objects. (b) Accuracy of predicting segmentation mask. (c) Loss value according to iteration.

**Figure S4.** Color change over time at minimum activation threshold: (a) **NPNR_2_**; (b) **NPBr**; (c) **NP31**; and (d) **NP52**.

**Figure S5**. Cyclic stability and recovery behavior of mechanophore-embedded PDMS samples under repeated FUS loading. Time-resolved normalized red, green, and blue intensity profiles are shown for **NPNR_2_**, **NPBr**, **NP31**, and **NP52** over three repeated measurements. All samples exhibited reproducible color changes during loading and gradual recovery after FUS removal. **NPBr** recovered more rapidly (~18 s), whereas **NPNR_2_** showed a slower recovery (~50 s). The mixed samples, **NP31** and **NP52**, also exhibited recovery times of ~50 s, indicating that their overall recovery behavior was dominated by **NPNR_2_**.

**Figure S6**. Representative optical images of **NPNR_2_**, **NPBr**, **NP31**, and **NP52** at selected time points (0 s, 10 or 20 s, and 80 s), corresponding to the RGB intensity profiles shown in Figure S5: (a) before normalization; and (b) after normalization to a non-responsive background region.

**Figure S7.** Spatiotemporal control of mechanical force using FUS: (a) Schematic of focused transducer geometry and ultrasonic beam pattern. The mechanical energy is concentrated within an ellipsoidal focal region defined by the beamwidth and depth of field ^[3]^, enabling precise spatial control. (b) Diagram of FUS operational parameters, including number of pulses, pulse length, repetition interval, duty cycle, and total sonication time. Temporal control of mechanical force is achieved by adjusting these parameters.

**Figure S8.** Preprocessing sequence for PTV ^[7]^: (a) The region of interest (ROI) is extracted from the original image, and the green channel is isolated to enhance bead contrast. Preprocessing steps including local mean filtering, Gaussian blurring ^[8]^, and frame stabilization ^[9]^ are applied to reduce noise and correct for drift caused by camera vibrations. Filtered images are binarized using Otsu’s method ^[10]^, and bead centroids are detected and tracked across frames using the Hungarian algorithm ^[11,12]^; and (b) Filtering significantly improves SNR and particle segmentation accuracy, enhancing the reliability of centroid detection.

**Figure S9.** Voltage-to-pressure conversion of the FUS ^[4]^. Input voltage from the function generator is detected as output voltage by the hydrophone, which is then converted to acoustic pressure using its sensitivity calibration data.

**Figure S10.** Color-activated regions predicted by Mask R-CNN for each sample across different energy intensity (I_SPTA_).

**Figure S11.** Stress-dependent variations in normalized RGB intensity for each sample.

**Figure S12.** Representative color changes before and after FUS activation for (a) **NPNR_2_**, (b) **NPBr**, (c) **NP31**, and (d) **NP52**, along with corresponding RGB intensity values. All RGB values are normalized to a non-activated background regions to account for imaging variability and facilitate accurate comparison of chromogenic response across samples. All RGB analyses in this study were performed using these normalized values.

**Figure S13**. Optical microscopy images of silver-coated microbead particles dispersed in the PDMS matrix: (a) Bright-field and (b) dark-field optical image, both showing a uniform particle distribution (inset: photograph of the PDMS mixture containing 0.015 wt% microbeads); and (c) A high-magnification dark-field image.

**Figure S14**. Control experiment using blank PDMS without mechanophores under FUS irradiation. Time-resolved normalized red, green, and blue intensity profiles were monitored during and after FUS exposure at 205 kg s^-2^ cm^-2^ for 20 s, the strongest loading condition used for **NP52**. Representative optical images at 0 s, 10 s, 20 s, and 30 s are shown alongside the RGB profiles. No noticeable change was observed in either the optical images or the RGB intensities, confirming that the PDMS matrix itself does not produce any measurable optical interference under FUS exposure.

**Figure S15**. Thermal control experiment for mechanophore-embedded PDMS samples: (a) schematic illustration of the temperature-controlled setup and the corresponding temperature profile; (b) representative optical images and normalized red, green, and blue intensities of **NPNR_2_**, **NPBr**, **NP31**, and **NP52** before heating (25 °C) and after heating (43 °C). All samples were heated to 43 ℃ in a temperature-controlled chamber, corresponding to the maximum temperature measured during FUS irradiation. Neither noticeable color change nor significant variation in RGB intensities was observed after heating, demonstrating that the temperature increase alone does not activate the mechanophores under the FUS conditions used in this study.

**Figure S16.** Color saturation of the NP52 sample under compressive stress using the MTS shown in Figure 6(c): (a) Compressive stress-time profile used to evaluate the time-dependent color response. The sample was repeatedly loaded to approximately 15.8 MPa with increasing holding times corresponding to points (1)-(5) and photographed immediately after unloading at each time point. Before each subsequent loading cycle, the sample was allowed to fully recover to its original color. (b) Normalized RGB intensities at the corresponding loading times, showing progressive color change followed by saturation under sustained compressive stress.

**Captions for Movies**

**Movie S1.** Mechanochromic response of **NPNR_2_**-embedded PDMS under FUS. A localized, circular region at the acoustic focal spot shows a reversible purple color change upon FUS activation, confirming repeatable mechanophore activation. The spatial confinement of the color change highlights the precision of the spatiotemporally released acoustic radiation force.

**Movie S2.** Mechanochromic response of **NPBr**-embedded PDMS under FUS. A localized, circular region at the acoustic focal spot shows a reversible yellow color change upon FUS activation, confirming repeatable mechanophore activation. The confined nature of the color change highlights the precision of the spatiotemporally released acoustic radiation force.

**Movie S3.** Mechanochromic response of **NP31**-embedded PDMS under FUS. A gradual reddish color shift emerges in the focal spot upon FUS activation, indicating the combined activation of both **NPNR_2_** (purple) and **NPBr** (yellow) mechanophores. The color reverts upon cessation of FUS, demonstrating reversible, multichannel mechanochromism.

**Movie S4.** Mechanochromic response of **NP52**-embedded PDMS under FUS. A clear reddish color change appears at the focal spot during FUS activation, corresponding to the sequential activation of both **NPNR_2_** (purple) and **NPBr** (yellow) mechanophores. The more intense color distribution compared to **NP31** reflects the higher **NPNR_2_** content. The response is fully reversible upon cessation of FUS, highlighting its suitability for repeatable, spatially confined stress sensing.

**Movie S5.** Strain evolution in **NPNR_2_**-embedded PDMS under FUS. Silver-coated microbeads embedded in the matrix exhibit inward displacement toward the focal center during FUS exposure (within the dashed circle), followed by gradual outward return upon cessation. This motion confirms reversible, localized strain with a peak magnitude of ~ 1.2 % and a full recovery time of ~ 9 s.

**Movie S6.** Strain evolution in **NPBr**-embedded PDMS under FUS. Upon FUS activation, silver-coated microbeads within the focal spot (within the dashed circle) exhibit pronounced inward displacement, corresponding to a higher peak strain of ~ 2.8 % stain than that observed in **NPNR_2_** due to the greater activation threshold of **NPBr**. The sample rapidly returns to its original state within ~ 2 s.

**Movie S7.** Strain evolution in **NP31**-embedded PDMS under FUS. Upon FUS exposure, silver-coated microbeads within the focal spot (within the dashed circle) exhibit inward displacement, corresponding to a peak strain of ~ 2.3%. The sample gradually returns to its initial configuration within ~ 7 s, indicating reversible strain behavior.

**Movie S8.** Strain evolution in **NP52**-embedded PDMS under FUS. Upon FUS application, silver-coated microbeads within the focal spot (within the dashed circle) undergo inward displacement, corresponding to a peak strain of ~ 2.2%. The strain reverses gradually, with full recovery occurring within ~6 s, indicating robust and reversible deformation behavior.

**References**

[1]	B. A. Versaw, M. E. McFadden, C. C. Husic, M. J. Robb, *Chem. Sci.* **2020**, *11*, 4525.

[2]	M. K. Beyer, *J. Chem. Phys.* **2000**, *112*, 7307.

[3]	K. Raum, W. D. O’Brien, *IEEE Trans. Ultrason. Ferroelectr. Freq. Control* **1997**, *44*, 810.

[4]	G. Kim, V. M. Lau, A. J. Halmes, M. L. Oelze, J. S. Moore, K. C. Li, *Proc. Natl. Acad. Sci. U. S. A.* **2019**, *116*, 10214.

[5]	X. Wang, G. Kim, J. L. Chu, T. Song, Z. Yang, W. Guo, X. Shao, M. L. Oelze, K. C. Li, Y. Lu, *J. Am. Chem. Soc.* **2022**, *144*, 5812.

[6]	K. He, G. Gkioxari, P. Dollár, R. Girshick, In *Proceedings of the IEEE international conference on computer vision*, **2017**, pp. 2961–2969.

[7]	E. A. Cowen, S. G. Monismith, E. A. Cowen, S. G. Monismith, *Exp. Fluids* **1997**, *22*, 199.

[8]	R. A. Haddad, A. N. Akansu, *IEEE Trans. Signal Process.* **1991**, *39*, 723.

[9]	Y. Shen, P. Guturu, T. Damarla, B. P. Buckles, K. R. Namuduri, *IEEE Trans. Consum. Electron.* **2009**, *55*, 1714.

[10]	N. Otsu, *Automatica* **1975**, *11*, 23.

[11]	F. Luetteke, X. Zhang, J. Franke, In *ROBOTIK 2012; 7th German Conference on Robotics*, VDE, **2012**, pp. 1–6.

[12]	J.-T. Kim, D. Kim, A. Liberzon, L. P. Chamorro, *J. Vis. Exp. JoVE* **2016**, 53745.
